# The Hypertension of Hemophilia Is Not Explained by the Usual Cardiovascular Risk Factors: Results of a Cohort Study

**DOI:** 10.1155/2016/2014201

**Published:** 2016-11-14

**Authors:** Richard F. W. Barnes, Thomas J. Cramer, Afrah S. Sait, Rebecca Kruse-Jarres, Doris V. K. Quon, Annette von Drygalski

**Affiliations:** ^1^Department of Medicine, Division of Hematology/Oncology, University of California San Diego, San Diego, CA, USA; ^2^Department of Medicine, University of British Columbia, Vancouver, BC, Canada; ^3^School of Medicine, Section of Hematology/Oncology, Tulane University, New Orleans, LA, USA; ^4^Washington Center for Bleeding Disorders at Bloodworks NW, Seattle, WA, USA; ^5^Orthopaedic Hemophilia Treatment Center, Orthopaedic Institute for Children, Los Angeles, CA, USA; ^6^Department of Molecular and Experimental Medicine, The Scripps Research Institute, La Jolla, CA, USA

## Abstract

*Background*. The etiology of the high prevalence of hypertension among patients with hemophilia (PWH) remains unknown.* Methods*. We compared 469 PWH in the United States with males from the National Health and Nutrition Examination Survey (NHANES) to determine whether differences in cardiovascular risk factors can account for the hypertension in hemophilia.* Results*. Median systolic and diastolic BP were higher in PWH than NHANES (*P* < 0.001) for subjects not taking antihypertensives. Those taking antihypertensives showed similar differences. Differences in both systolic and diastolic BP were especially marked among adults <30 years old. Differences between PWH and NHANES persisted after adjusting for age and risk factors (body mass index, renal function, cholesterol, smoking, diabetes, Hepatitis C, and race).* Conclusions*. Systolic and diastolic BP are higher in PWH than in the general male population and especially among PWH < 30 years old. The usual cardiovascular risk factors do not account for the etiology of the higher prevalence of hypertension in hemophilia. New investigations into the missing link between hemophilia and hypertension should include age of onset of hypertension and hemophilia-specific morbidities such as the role of inflammatory joint disease.

## 1. Introduction

About 1 in 5,000 male births in the United States results in hemophilia which is an X-linked bleeding disorder. In the 19th century, hemophilia was a rare disease in adulthood, with a median life expectancy of 11 years. Life expectancy increased when clotting factors were developed in the 1960s, with many patients surviving into middle age and beyond [1, 2]. The emergence of HIV in the early 1980s dramatically increased mortality among patients because the virus was disseminated in blood products. Today, with the advent of virally safe clotting factor preparations in the early 1990s, life expectancy approaches that of males in the general population [2, 3]. This has unmasked new comorbidities, such as the hypertension in hemophilia, that are incompletely characterized and poorly understood [4–8].

Bleeding in hemophilia most frequently manifests as spontaneous joint and muscle bleeding, resulting in progressive joint degradation. The most serious complications are intracranial hemorrhages (ICH) which are 20 to 50 times more frequent in patients with hemophilia (PWH) compared to the general male population [9] with a mortality rate up to 20% [10–12]. Hypertension is a major risk factor for ICH because the risk of ICH increases steeply with the stage of hypertension [9–11, 13, 14]. This is of particular clinical concern since there is increasing evidence that hypertension is more common in PWH compared to the general population [4–8, 15]. The reasons for the higher prevalence of hypertension in PWH remain obscure.

Hypertension in the general population is associated with age, BMI, cholesterol, kidney function, diabetes, smoking, HCV, and race [16]. Age, BMI, and diabetes were correlated with hypertension in PWH in two studies [6, 7], and renal function was inversely associated in one [7]. If hypertension is more prevalent among PWH than the general population, then it is reasonable to assume that one or more risk factors must be higher in PWH than among the general population. Alternatively, higher blood pressure among PWH could be explained if it increases more rapidly with a particular risk factor—the slope of blood pressure on the risk factor is steeper—than for other males. Our objective was to analyze the association between blood pressure and each of the usual risk factors to determine whether one or more risk factors could account for the hypertension of hemophilia.

Hypertension is a categorical variable that is derived from measurements of blood pressure (BP). We chose to analyze systolic and diastolic BP measurements, which are continuous variables and therefore provide greater statistical power [17]. We examined BP trends in relation to the usual cardiovascular risk factors by comparing a cohort of PWH against a randomly selected sample of males from the population of the United States. While previous studies have compared systolic BP or prevalence of hypertension against the general population [6, 8] or compared BP against the general male population adjusted for age in the Netherlands [5], this study focuses on a comparison of BP values adjusted for age between PWH and the general male population of the United States. In addition, we analyzed subjects treated with antihypertensive medications separately from untreated subjects.

## 2. Methods

### 2.1. Patients with Hemophilia

A retrospective data collection was performed for all male patients with hemophilia (PWH) aged 18 years and older seen regularly at three hemophilia treatment centers in the United States: University of California San Diego (2004–2014), Tulane University (2008–2011), and the Los Angeles Orthopaedic Hospital (2005–2012). Patient confidentiality safeguards and data acquisition methods were approved by the Institutional Review Boards of all three institutions. Only patients with complete data on age and race (Hispanic, white, black, and other) were included (*n* = 469). Data extracted included demographic information on age, ethnicity, hemophilia type and severity, positive tests for hepatitis C (HCV) or HIV by serology or reported history thereof, medication history, and smoking status.

Laboratory values of nonfasting patients were obtained during regular clinic visits. Data pertaining to diabetes (HbA1c, random blood glucose) and serum creatinine were recorded. The diagnosis of diabetes was defined according to the 2010 American Diabetes Association Standards of Medical Care in Diabetes as medication use for glycemic control, HbA1c > 6.5, or presence of ≥ 2 random glucose levels above 200 mg/dL [18]. Age, BMI (kg/m^2^), and creatinine were recorded at the patient's final clinic visit. Renal function was determined by estimated glomerular filtration rate (eGFR) calculated using the CKD-EPI equation [19].

Blood pressure in all clinics was measured in accordance with the current recommendations of the American Heart Association [20]. In brief, blood pressures were obtained by licensed staff using calibrated automated manometers with subjects in a chair at rest, arm supported at heart level. The 3 most recent blood pressure measurements were used for analysis (the mean number of measurements was 2.6, 2.4, and 2.9 for University of California San Diego, Tulane University, and Los Angeles Orthopaedic Hospital, resp.).

PWH were divided into two groups: treated (those taking antihypertensive medications, *n* = 118) and untreated (those not taking such medications, *n* = 342).

### 2.2. Control Population

We compared the PWH to males drawn from the adult population of the United States during National Health and Nutrition Examination Surveys (NHANES). This is a series of surveys to evaluate the health status of the nation [21]. The data are freely available to the public (http://www.cdc.gov/nchs/nhanes/index.htm). Three cycles of NHANES (2007-2008, 2009-2010, and 2011-2012) were combined to give a large sample.

The age distribution of patients suffering from severe hemophilia is underrepresented in the older ages, and blood pressure varies with age as well as by race [16]. Therefore, in order to ensure comparable age distributions, we randomly selected untreated NHANES subjects to match untreated PWH by race (Hispanic, white, black, and other) and age-class (18–29, 30–39, 40–49, 50–59, 60–69, and 70–79 years) in the ratio of 5 NHANES subjects to each PWH. Similarly, for treated PWH we selected a comparison group of treated NHANES subjects in the same manner. Because there were few NHANES subjects under 40 being treated for hypertension, the actual ratio for treated subjects was 4.6 NHANES to each PWH.

### 2.3. Statistical Methods

The values of SBP, DBP, and continuous covariates were shown as medians and interquartile ranges because most were not normally distributed. Their values were compared by Wilcoxon tests. Categorical variables were compared by *χ* ^2^ tests.

We used analysis of covariance to test the proposition that the difference between the BP of PWH and NHANES subjects was due to one of the risk factors [22]. The outcome variables were log SBP and DBP. We used log SBP because it gave normally distributed residuals while SBP resulted in skewed residuals.

A binary variable *Z* distinguished NHANES subjects [*Z* = 0] and PWH [*Z* = 1]. The covariates were BMI, creatinine, glomerular filtration rate (eGFR), total cholesterol, diabetes (yes/no), HCV (yes/no), HIV (yes/no), smoking (never/former/current), and race (Hispanic, white, black, and other). HIV was dropped because too few NHANES subjects were positive. BMI and total cholesterol were transformed to logs and creatinine to log (1 + creatinine).

The BP analyses were run separately for young adults (18–29 years) and older adults (30–79 years) because exploratory analyses had shown differences between them in the associations with some covariates.

For each covariate *X* the regression model was log SBP on *X*, *Z*, age, and the interaction *X∗Z*. When DBP was the outcome we used the quadratic form of age [23] with centered age,* Cage*, to avoid multicollinearity [22]. Thus the regression model was DBP on *X*, *Z*,* Cage*,* Cage* ^*2*^, and *X∗Z*. If *P* < 0.10 for *X∗Z* interaction then an analysis of covariance was not possible because the slopes for NHANES subjects and PWH were not parallel [22]. If *P* > 0.10 for that interaction then the slopes were assumed to be equal and an analysis of covariance tested the hypothesis that the mean of the outcome, after adjustment for *X* and age, did not differ between PWH and NHANES subjects. That is, if the regression coefficient for *Z* differed from zero then we can assume that this particular risk factor could not account for the difference in BP between PWH and NHANES subjects. On the other hand, if the regression coefficient did not differ from zero then the higher BP value for PWH may be due to that particular risk factor.

Finally, having tested each covariate individually, we ran a full model with all covariates to test the proposition that these risk factors in combination explain the difference between PWH and NHANES subjects. Again, all interactions between *Z* and each covariate were tested. If it appeared that a particular covariate *X* caused the difference between PWH and NHANES subjects to disappear, then we also ran the full model without *X* in order to observe the effect of all risk factors with and without *X*.

## 3. Results

### 3.1. Demographics

There were 469 PWH of whom four-fifths had hemophilia A and 56% suffered from the severe form (see Table 1). About half were white and a quarter Hispanic.

### 3.2. Blood Pressures and Cardiovascular Risk Factors

**Figure 1:**
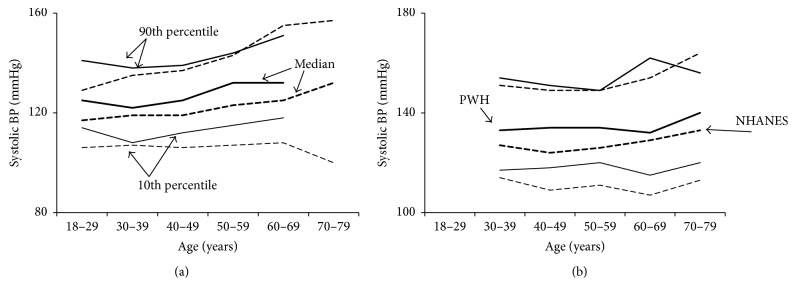
Systolic blood pressure as a function of age. Systolic blood pressure was higher among patients with hemophilia (PWH) compared to men of the general United States population (NHANES) at all ages, whether or not the subjects were taking antihypertensive medications. PWH are shown by solid lines and NHANES subjects by broken lines. Tenth percentiles, medians, and 90th percentiles are shown. The lower percentile lines show the 10th, while the upper percentile lines show the 90th. (a) Systolic blood pressure for untreated subjects. (b) Systolic blood pressure for treated subjects (taking antihypertensive medications).

**Figure 2:**
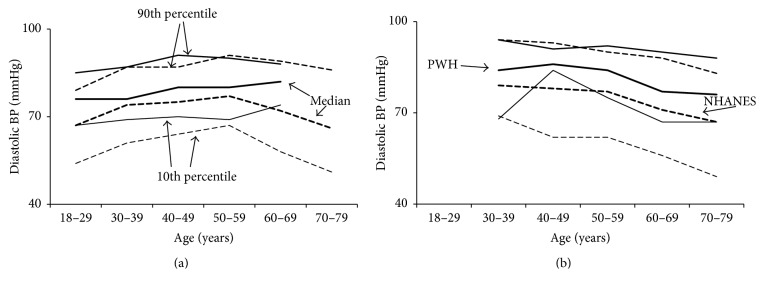
Diastolic blood pressure as a function of age. Diastolic blood pressure was higher among patients with hemophilia (PWH) compared to men of the general United States population (NHANES) at all ages, whether or not the subjects were taking antihypertensive medications. PWH are shown by solid lines and NHANES subjects by broken lines. Tenth percentiles, medians, and 90th percentiles are shown. The lower percentile lines show the 10th, while the upper percentile lines show the 90th. (a) Diastolic blood pressure for untreated subjects. (b) Diastolic blood pressure for treated subjects (taking antihypertensive medications).

Both SBP and DBP values were significantly higher among PWH compared to NHANES subjects, whether subjects were taking antihypertensives or not. In PWH not taking antihypertensives median SBP and DBP were 125 and 78 mmHg (118 and 72 mmHg in NHANES) (Table 2), and in PWH taking antihypertensives SBP and DBP were 134 and 84 (127 and 76 mmHg in NHANES), respectively (Table 3); all *P* < 0.001. While PWH and NHANES subjects showed similar trends in BP by age, PWH had higher median BP values and 10th percentiles (Figures 1 and 2). The difference in 10th percentiles was particularly marked for DBP among treated PWH.

Despite their higher BP levels, PWH had better risk profiles, with lower BMI and total cholesterol and better renal function as reflected by creatinine and eGFR levels (Tables 2 and 3). Fewer PWH had ever smoked, and there were fewer diabetics among the PWH. On the other hand, as a consequence of virally contaminated blood products in earlier years, the prevalence of HCV and HIV was much higher.

In the regression models with adjustment only for age, log SBP and DBP remained higher among PWH than NHANES subjects, whether they were treated or untreated (Tables 4 [Table tab5] [Table tab6]–7). When adjusting for age, BMI, renal function, cholesterol, smoking, diabetes, HCV, or race, only HCV emerged as a potential explanation for the higher BP values among PWH and then only for untreated subjects over 30 (Tables 4 and 6). The difference in BP between PWH and NHANES subjects was especially marked among the younger adults (Tables 4 and 6).

#### 3.2.1. SBP for Untreated Subjects

**Figure 3:**
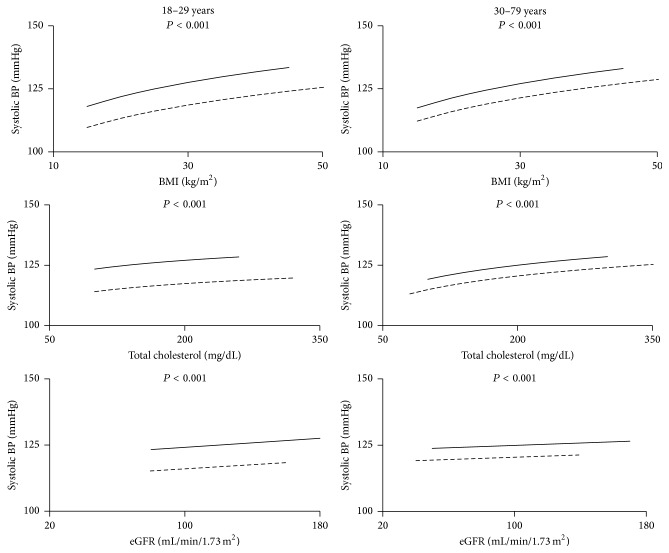
Systolic blood pressure for untreated subjects as a function of BMI, cholesterol, and estimated glomerular filtration rate (eGFR). The solid lines are the regression lines for PWH while the broken lines are for NHANES subjects. The *P* value for the difference between PWH and NHANES is shown.

The comparison between SBP curves for PWH and NHANES subjects illustrated that the difference between curves is much greater for young adults (18–29 years) compared to older adults (≥30 years) (Figure 3).

For young adults, after adjusting for age and each covariate alone (BMI, renal function, cholesterol, smoking, diabetes, HCV, and race) and then for all covariates together, the difference in SBP between PWH and NHANES subjects remained (Table 4). Similarly, the difference in SBP between PWH and NHANES subjects remained for subjects ≥ 30 years after adjusting for each of age, BMI, creatinine, eGFR, cholesterol, smoking, or race (Figure 3 and Table 4). However, in contrast to the young adults, the difference disappeared when the model was adjusted for HCV. When all covariates together were in the full model there was no difference between PWH and NHANES. When HCV was removed from the full model the difference reappeared, indicating that among the tested risk factors it was only HCV that might explain the difference in systolic BP among older adults not taking antihypertensives.

#### 3.2.2. SBP for Treated Subjects

**Figure 4:**
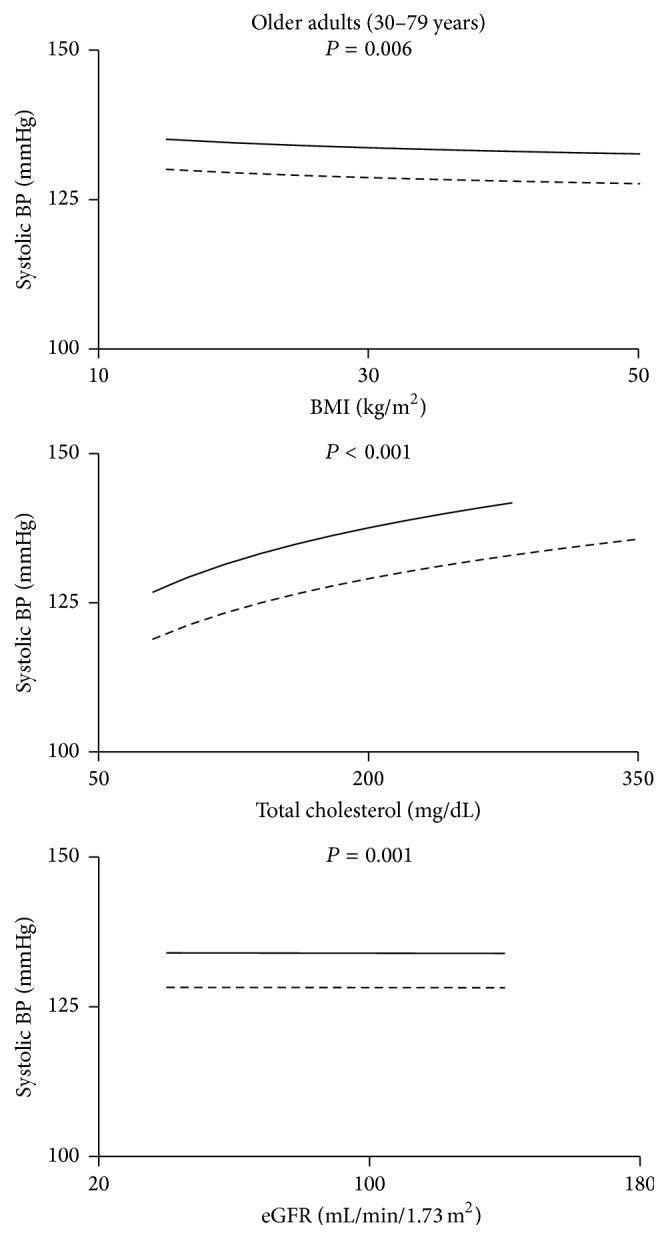
Systolic blood pressure for subjects treated with antihypertensive medications as a function of BMI, cholesterol, and estimated glomerular filtration rate (eGFR). The solid lines are the regression lines for PWH while the broken lines are for NHANES subjects. The *P* value for the difference between PWH and NHANES is shown.

There were too few subjects in the young age group taking antihypertensive medications (*n* = 5 PWH) to justify analysis. For older adults taking antihypertensives the difference in SBP between PWH and NHANES subjects persisted after adjusting for each covariate alone and with all covariates together (Figure 4 and Table 5).

#### 3.2.3. DBP for Untreated Subjects

**Figure 5:**
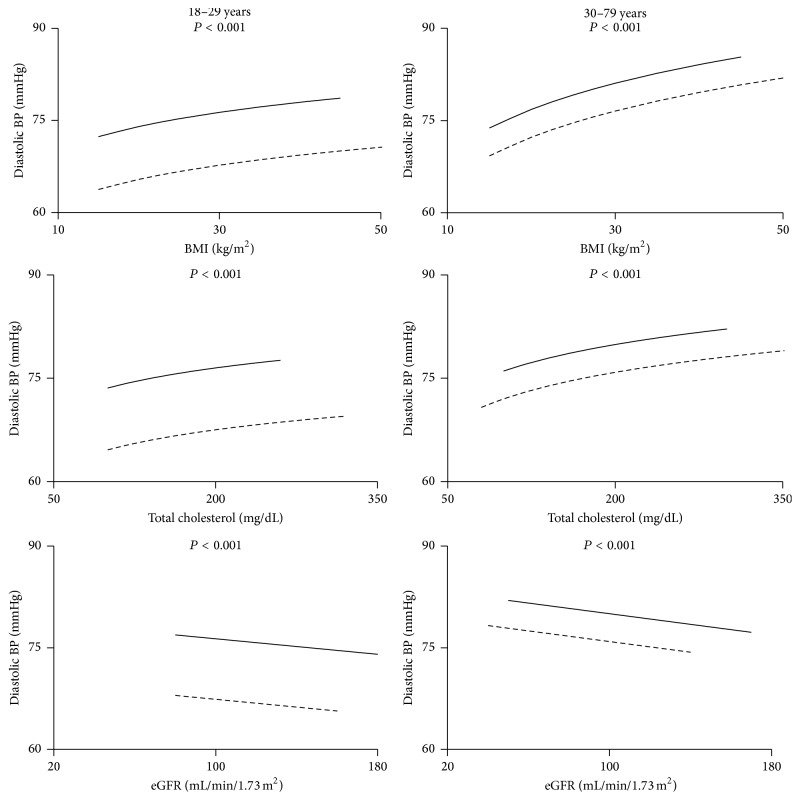
Diastolic blood pressure for untreated subjects as a function of BMI, cholesterol, and estimated glomerular filtration rate (eGFR). The solid lines are the regression lines for PWH while the broken lines are for NHANES subjects. The *P* value for the difference between PWH and NHANES is shown.

After adjusting for age and each covariate alone and then for all covariates together, the difference between PWH and NHANES remained for young adults (18–29 years) (Figure 5 and Table 6). Also, the difference between PWH and NHANES remained for subjects ≥ 30 years after adjusting for age, BMI, creatinine, eGFR, cholesterol, or smoking. However, as seen above with SBP, adjusting for HCV reduced the difference for subjects ≥ 30 years (Table 6). The interaction between *Z* (the binary variable that distinguished NHANES subjects from PWH) and race shows that the difference in DBP between PWH and NHANES subjects also depended upon race: the largest differences were among whites whereas black, Hispanic, and “other” did not show differences between PWH and NHANES (Table 8). When all covariates were included (the full model) the differences between PWH and NHANES for blacks, Hispanics, and “other” disappeared, but not for whites. The differences between PWH and NHANES reappeared when HCV was removed from that model (Table 9), indicating that among the tested risk factors it was only HCV that might explain the difference in diastolic BP among older adults not taking antihypertensives.

#### 3.2.4. DBP for Treated Subjects

**Figure 6:**
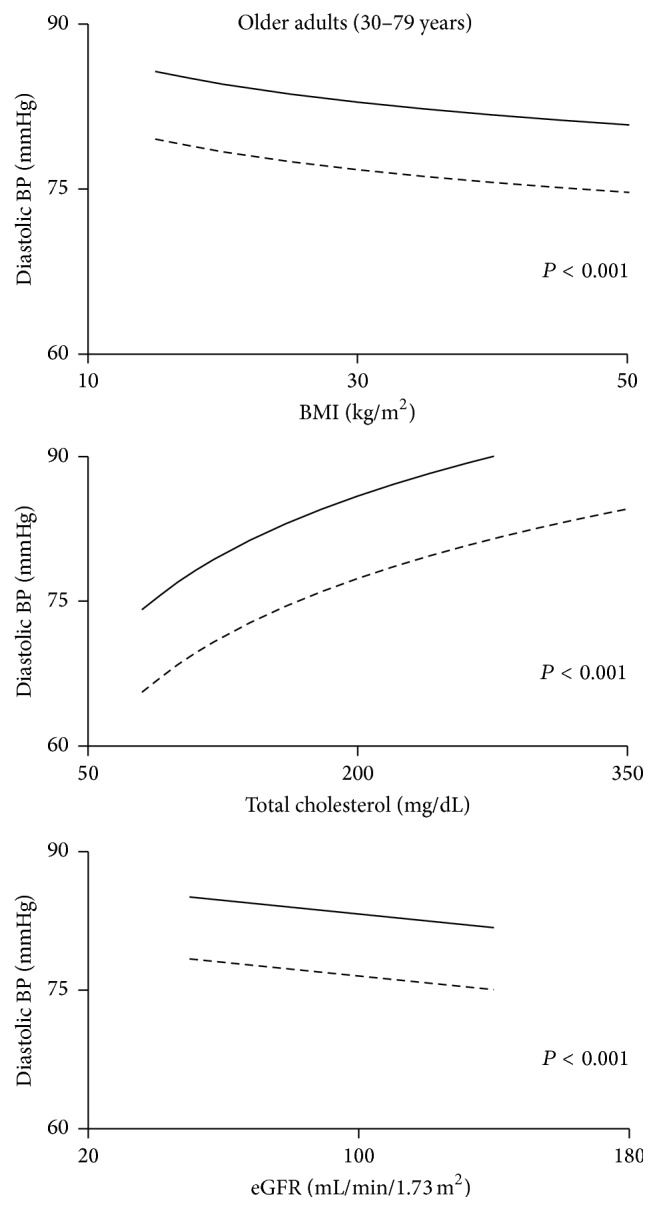
Diastolic blood pressure for subjects treated with antihypertensive medications as a function of BMI, cholesterol, and estimated glomerular filtration rate (eGFR). The solid lines are the regression lines for PWH while the broken lines are for NHANES subjects. The *P* value for the difference between PWH and NHANES is shown.

There were too few subjects in the young age group taking antihypertensive medications (*n* = 5 PWH) to justify analysis. For subjects ≥ 30 years, the difference between PWH and NHANES subjects persisted after adjusting for each covariate alone (Figure 6 and Table 7) and with all covariates together.

## 4. Discussion

### 4.1. Blood Pressure Trends

Here we report the results of a large cohort study showing that the usual cardiovascular risk factors do not account for the higher blood pressure readings in hemophilia patients. We examined BP measurements while adjusting for age because the causes of hypertension are more likely to be revealed by examining its components—the two BP variables—than by examining hypertension itself. Furthermore, using continuous variables as outcomes is more likely to bring relationships with risk factors to light than if we were to use a binary outcome like hypertension [17].

This is the first study to examine systolic and diastolic measurements for PWH in separate treated (for antihypertensive medications) and untreated categories. PWH showed higher systolic and diastolic measurements than the general male population no matter which risk factor was included in the model.

Blood pressure measurements were higher for PWH even for subjects treated with antihypertensives. These observations imply that PWH are less responsive to such drugs, assuming comparable adherence to medications in both groups. However, since the treatment variable for PWH was based on charted use of medication, some patients may not have used the drugs as prescribed, while NHANES was self-reported drug use.

Although the 90th percentiles for PWH were similar to those for NHANES subjects, their 10th percentiles were higher. This indicates that the frequency distribution of BP covers a narrower range for PWH, with similar maxima but fewer low values. Among PWH the BP did not fall as much in diastole as among NHANES subjects, suggesting greater stiffness of the vascular walls. This phenomenon was particularly noticeable in treated subjects. These observations are new and are consistent with previous observations describing impaired flow-mediated vessel dilation and decreased vascular endothelial function among PWH [24]. Thus PWH may suffer from systemic vascular changes that require further investigation. Towards that end, vascular remodeling in joints with evidence for systemic mediation was recently described as a unique feature of hemophilia [25], indicating vascular abnormalities of uncertain etiology that may be linked to the hypertension in PWH.

An unexpected finding was the marked elevated BP of the youngest age group, which is an alarming and worrisome finding, warranting future investigations in youth and children with hemophilia. With the exception of results from a small cohort study in the 1980s that found increased BP values in PWH [5], contemporary information regarding BP trends in PWH on a larger scale does not yet exist. Consequently, results from this study contribute important new information.

### 4.2. Risk Factors for Elevated Blood Pressures

Next, we examined whether one or more cardiovascular risk factors could explain the higher BP in PWH. First, one would expect to find at least one of the risk factors for high BP to be elevated in PWH. However, the converse was the case: PWH weighed less and had lower serum cholesterol, their serum creatinine levels and eGFR levels showed better kidney function, and they had lower rates of diabetes and smoking. Others have also found PWH to have better weight and cholesterol profiles [5, 26, 27], although one large study of PWH also noted that their diabetes and smoking rates were similar to the general population [26].

Second, BP levels were positively associated with cardiovascular risk factors such as age, BMI, and cholesterol, as would be expected. However the BP curves for PWH and NHANES subjects rose in parallel, and for all risk factors the BP curve for PWH was always above that for NHANES subjects. Higher BP among PWH cannot be explained by a steeper slope than for NHANES. Therefore, none of the usual cardiovascular risk factors could explain the higher BP in PWH.

Hypertension is well known to be one of the many consequences of HCV infection that include hyperglycemia, insulin resistance, diabetes mellitus, left ventricular mass index, disturbed lipid metabolism, endothelial dysfunction, inflammation, and vessel damage as well as greater vascular stiffness [28–33]. It is therefore not surprising that HCV appeared to contribute to some extent to higher BP. Interestingly, HCV only explained the higher BP in untreated PWH over 30 years. Subjects in this category infected with HCV had higher BP values than uninfected individuals after adjusting for other covariates. However, HCV did not explain higher BP among treated PWH where HCV prevalence was high (82.2% in PWH compared to 3.4% in NHANES subjects) or in young patients, where HCV prevalence was lower (36.4% in PWH compared to 0.5% in NHANES subjects). Thus, for PWH the associations between BP and HCV varied with both age and treatment status and possibly with race. While HCV infection may contribute to higher BP in PWH, it does not fully explain the differences between PWH and NHANES.

The association between hypertension and insulin resistance (IR) remains obscure [34, 35]. Possibly a third factor may promote both IR and hypertension; for example, catecholamines are implicated in both carbohydrate metabolism and vascular resistance [35–37]. We could not examine the association between the homeostatic model assessment (HOMA) index, which is a measure of IR, and blood pressure because insulin concentrations were not measured in our cohort of PWH. However, the lower prevalence of diabetes among PWH suggests that IR may be less common compared to NHANES. Nevertheless, IR and its association with HCV should be examined in future studies of hypertension in PWH.

There is a long-standing debate whether PWH have poor renal function, possibly linked to renal bleeding [38–40]. We specifically examined this topic since hypertension is associated with poorer renal function in the general population [16]. As previously observed [7], PWH had superior renal function despite having higher BP. This reinforces the argument that elevated BP levels in hemophilia are largely independent of the usual cardiovascular risk factors. However, since some studies have not found associations between hypertension and renal function in PWH [5, 6, 15], the questions concerning renal function, renal bleeding, and hypertension in hemophilia remain unresolved.

This study, like several others [4–6], was limited by the small number of patients because hemophilia is a rare disease. However, it contributes significantly to the accumulating evidence that the hypertension of hemophilia is an important comorbidity. Also, several other limitations have to be mentioned. First, BP measurements for NHANES subjects were taken on one day while PWH had frequent follow-up [26]. On the other hand, NHANES subjects with their single measurements outnumbered PWH by 5 : 1 and the large differences between PWH and NHANES subjects at all ages are strong evidence for higher values in hemophilia. Second, BP measurements were made by a large number of different people using a variety of equipment. It is unlikely that one group (PWH or NHANES) would have a systematic bias in one direction. But the variation in measurements is greater than if each group were measured by one provider using a single instrument, and that would reduce the power of statistical tests. Third, our regression models accounted for only a small proportion of the variance in SBP and DBP: much of the variation in BP for both PWH and NHANES subjects remains unexplained. Fourth, while HCV may explain some of the difference, we must be cautious since few NHANES subjects were infected.

## 5. Conclusion

We examined systolic and diastolic blood pressure in subjects that were divided into those taking or not taking antihypertensive medications. This study demonstrates that PWH suffer from higher BP levels than the general male population at all ages whether or not they are treated for hypertension. Further, their elevated BP levels cannot be easily explained by the usual cardiovascular risk factors. From a pragmatic clinical standpoint these findings are important since care paradigms for PWH need to develop a stronger focus on BP control to avoid the risk of mortality from ICH [9–12]. These findings are also important from a basic scientific standpoint: the etiology of the “Hypertension of Hemophilia” remains largely unresolved. Abnormal vascular stiffness and vascular remodeling in joints have been described in hemophilia [24, 25] and may be mediated systemically and are possibly related to the hypertension. Also, higher systolic and diastolic values seen among PWH in their twenties highlight questions about age of onset and, again, underlying etiology. New studies will be required to unravel the interrelations of vascular abnormalities, BP, and age of onset of hypertension among PWH.

## Figures and Tables

**Table 1:** Demographics for the patients with hemophilia (*n* = 469).

		Frequency	
		*n*	%
Race	White	249	53.1
	Black	53	11.3
	Hispanic	115	24.5
	Other	52	11.1
Hemophilia type	A	371	79.1
	B	97	20.7
	Unknown	1	0.2
Hemophilia severity	Severe	263	56.1
	Moderate	65	13.9
	Mild	139	29.6
	Unknown	2	0.4
Inhibitor	Positive	28	6.0
	Negative	402	85.7
	Not tested	39	8.3

**(a):** 

Variable	NHANES		PWH		*P*
	Median (IQR)	*n*	Median (IQR)	*n*	
Systolic BP (mmHg)	118 (112–125)	1710	125 (118–134)	342	<0.001
Diastolic BP (mmHg)	72 (64–78)	1710	78 (73–83)	342	<0.001
BMI (kg/m^2^)	26.9 (23.7–30.5)	1703	26.1 (22.9–29.3)	323	0.016
Creatinine (mg/dL)	0.92 (0.82–1.02)	1613	0.90 (0.75–1.00)	326	<0.001
eGFR (mL/min/1.73 m^2^)	107 (94–119)	1613	112 (100–124)	326	<0.001
Cholesterol (total) (mg/dL)	188 (162–217)	1618	170 (146–196)	205	<0.001

**(b):** 

Variable		NHANES	PWH	*P*
		*n* (%)	*n* (%)	
HIV	Positive	7 (0.5)	66 (20.5)	<0.001
	Negative	1464 (99.5)	256 (79.5)	
HCV	Positive	30 (1.9)	208 (63.8)	<0.001
	Negative	1590 (98.2)	118 (36.2)	
Diabetes	Positive	100 (5.9)	5 (1.5)	0.001
	Negative	1610 (94.2)	336 (98.5)	
Smoking	Current	473 (30.8)	42 (18.1)	<0.001
	Former	300 (19.5)	37 (16.0)	
	Never	765 (49.7)	153 (66.0)	

NHANES indicates subjects from the National Health and Nutrition Examination Survey of the general United States population; PWH, patients with hemophilia; IQR, interquartile range; BP, blood pressure; BMI, body mass index; eGFR, estimated glomerular filtration rate; and HCV, hepatitis C virus.

**(a):** 

Variable	NHANES		PWH		*P*
	Median (IQR)	*n*	Median (IQR)	*n*	
Systolic BP (mmHg)	127 (119–140)	539	134 (126–141)	118	<0.001
Diastolic BP (mmHg)	76 (67–83)	539	84 (76–89)	118	<0.001
BMI (kg/m^2^)	30.6 (27.3–35.0)	534	27.7 (24.8–31.0)	115	<0.001
Creatinine (mg/dL)	0.99 (0.86–1.12)	508	0.90 (0.80–1.10)	116	0.007
eGFR (mL/min/1.73 m^2^)	89 (73–101)	508	98 (74–108)	116	0.005
Cholesterol (total) (mg/dL)	186 (160–214)	509	164 (137–193)	85	<0.001

**(b):** 

Variable		NHANES	PWH	*P*
		*n* (%)	*n* (%)	
HIV	Positive	2 (0.7)	33 (29.5)	<0.001
	Negative	281 (99.3)	79 (70.5)	
HCV	Positive	17 (13.4)	91 (81.3)	<0.001
	Negative	491 (96.7)	21 (18.8)	
Diabetes	Positive	178 (33.0)	31 (26.3)	0.188
	Negative	361 (67.0)	87 (73.7)	
Smoking	Current	109 (20.2)	16 (18.6)	0.166
	Former	192 (35.6)	23 (26.7)	
	Never	238 (44.2)	47 (54.7)	

NHANES indicates subjects from the National Health and Nutrition Examination Survey of the general United States population; PWH, patients with hemophilia; IQR, interquartile range; BP, blood pressure; BMI, body mass index; eGFR, estimated glomerular filtration rate; and HCV, hepatitis C virus.

**Table 4:** Analyses of covariance comparing log SBP values of untreated (not taking antihypertensive medications) PWH and NHANES subjects after adjusting for age and each covariate.

Covariate	Regression coefficient for *Z* (PWH versus NHANES)			*r* ^2^
	*b*	95% CI	*P*	
Young adults (18–29 years)				
No covariates (age + *Z* only)	0.069	0.050, 0.088	<0.001	0.085
log BMI	0.073	0.058, 0.088	<0.001	0.177
log (1 + Creatinine)	0.068	0.052, 0.084	<0.001	0.110
eGFR	0.068	0.052, 0.084	<0.001	0.110
log TotalCholesterol	0.079	0.060, 0.098	<0.001	0.094
Smoking status	0.071	0.052, 0.089	<0.001	0.087
HCV	0.066	0.048, 0.085	<0.001	0.104
Race	0.068	0.053, 0.083	<0.001	0.101
All covariates^*∗*^	0.081	0.057, 0.105	<0.001	0.159

Older adults (30–79 years)				
No covariates (age + *Z* only)	0.031	0.013, 0.049	<0.001	0.041
log BMI	0.046	0.030, 0.061	<0.001	0.087
log (1 + Creatinine)	0.038	0.022, 0.054	<0.001	0.052
eGFR	0.037	0.020, 0.053	<0.001	0.052
log TotalCholesterol	0.036	0.017, 0.056	<0.001	0.060
Smoking status	0.035	0.017, 0.052	<0.001	0.043
HCV	0.012	−0.015, 0.039	0.384	0.051
Race	0.038	0.022, 0.053	<0.001	0.055
All covariates^*∗*^	0.005	−0.027, 0.036	0.769	0.121
All covariates except HCV	0.049	0.029, 0.069	<0.001	0.111

*Z* indicates the binary variable that distinguishes PWH from NHANES (*Z* = 1 for PWH, *Z* = 0 for NHANES); PWH, patients with hemophilia; NHANES, subjects from the National Health and Nutrition Examination Survey of the general United States population; *b*, regression coefficient for *Z* representing change in log SBP per unit increase of the selected covariate; CI, confidence interval; *r* ^2^, square of the multiple correlation coefficient; BMI, body mass index; eGFR, estimated glomerular filtration rate; HCV, hepatitis C virus.

^*∗*^Age, log BMI, eGFR, log TotalCholesterol, smoking status, HCV, and race.

**Table 5 tab5:** Analyses of covariance comparing log SBP values of treated (taking antihypertensive medications) PWH and NHANES subjects after adjusting for age and each covariate.

Covariate	Regression coefficient for *Z* (PWH versus NHANES)			*r* ^2^
	*b*	95% CI	*P*	
Older adults (30–79 years)				
No covariates (age + *Z* only)	0.047	0.017, 0.078	0.003	0.020
log BMI	0.038	0.011, 0.065	0.006	0.022
log (1 + Creatinine)	0.045	0.019, 0.072	<0.001	0.025
eGFR	0.044	0.018, 0.070	0.001	0.023
log TotalCholesterol	0.064	0.033, 0.095	<0.001	0.050
Diabetes	0.042	0.016, 0.068	0.001	0.024
Smoking status	0.042	0.012, 0.072	0.006	0.027
HCV	0.046	0.002, 0.090	0.039	0.023
Race	0.041	0.015, 0.066	0.002	0.041
All covariates^*∗*^	0.053	0.001, 0.104	0.045	0.087

*Z* indicates the binary variable that distinguishes PWH from NHANES (*Z* = 1 for PWH, *Z* = 0 for NHANES); PWH, patients with hemophilia; NHANES, subjects from the National Health and Nutrition Examination Survey of the general United States population; *b*, regression coefficient for *Z* representing change in log SBP per unit increase of the selected covariate; CI, confidence interval; *r* ^2^, square of the multiple correlation coefficient; BMI, body mass index; eGFR, estimated glomerular filtration rate; HCV, hepatitis C virus.

^*∗*^Age, log BMI, eGFR, log TotalCholesterol, diabetes, smoking status, HCV, and race.

**Table 6 tab6:** Analyses of covariance comparing DBP values of untreated (not taking antihypertensive medications) PWH and NHANES subjects after adjusting for age and each covariate.

Covariate	Regression coefficient for *Z* (PWH versus NHANES)			*r* ^2^
	*b*	95% CI	*P*	
Young adults (18–29 years)				
No covariates (*Cage* + *Cage* ^2^ + *Z* only)	9.03	6.80, 11.25	<0.001	0.113
log BMI	8.59	6.80, 10.38	<0.001	0.167
log (1 + Creatinine)	8.91	7.06, 10.76	<0.001	0.159
eGFR	8.92	7.08, 10.77	<0.001	0.159
log TotalCholesterol	8.97	6.66, 11.28	<0.001	0.123
Smoking status	8.28	6.13, 10.43	<0.001	0.121
HCV	8.18	6.01, 10.35	<0.001	0.156
Race	8.52	6.77, 10.27	<0.001	0.162
All covariates^*∗*^	10.38	7.43, 13.33	<0.001	0.163

Older adults (30–79 years)				
No covariates (*Cage* + *Cage* ^2^ + *Z* only)	2.99	1.21, 4.77	0.001	0.035
log BMI	4.54	3.04, 6.03	<0.001	0.083
log (1 + Creatinine)	4.18	2.62, 5.73	<0.001	0.049
eGFR	4.10	2.54, 5.65	<0.001	0.047
log TotalCholesterol	4.03	2.14, 5.91	<0.001	0.050
Smoking status	3.15	1.43, 4.87	<0.001	0.039
HCV	2.45	−0.14, 5.04	0.064	0.045
Race	^†^			0.062
All covariates^*∗*^	^†^			0.121
All covariates except HCV^*∗*^	^†^			0.116

*Z*  indicates the binary variable that distinguishes PWH from NHANES (*Z* = 1 for PWH, *Z* = 0 for NHANES); PWH, patients with hemophilia; NHANES, subjects from the National Health and Nutrition Examination Survey of the general United States population; *b*, regression coefficient for *Z* representing change in log SBP per unit increase of the selected covariate; CI, confidence interval; *r* ^2^, square of the multiple correlation coefficient; BMI, body mass index; eGFR, estimated glomerular filtration rate; HCV, hepatitis C virus.

^*∗*^ *Cage*, *Cage* ^2^, log BMI, eGFR, log TotalCholesterol, smoking status, HCV, and race.

^†^ *Z∗*race interaction was significant and therefore ANCOVA cannot be performed.

**Table 7:** Analyses of covariance comparing DBP values of treated (taking antihypertensive medications) PWH and NHANES subjects after adjusting for age and each covariate.

Covariate	Regression coefficient for *Z* (PWH versus NHANES)			*r* ^2^
	*b*	95% CI	*P*	
Older adults (30–79 years)				
No covariates (*Cage* + *Cage* ^2^ + *Z* only)	6.40	3.75, 9.05	<0.001	0.165
log BMI	6.13	3.79, 8.47	<0.001	0.175
log (1 + Creatinine)	6.92	4.62, 9.21	<0.001	0.168
eGFR	6.73	4.45, 9.00	<0.001	0.172
log TotalCholesterol	8.53	5.97, 11.10	<0.001	0.224
Diabetes	6.60	4.34, 8.85	<0.001	0.179
Smoking status	6.57	3.95, 9.19	<0.001	0.164
HCV	6.97	3.13, 18.81	<0.001	0.170
Race	6.68	4.42, 8.95	<0.001	0.175
All covariates^*∗*^	6.68	2.26, 11.10	0.003	0.239

*Z* indicates the binary variable that distinguishes PWH from NHANES (*Z* = 1 for PWH, *Z* = 0 for NHANES); PWH, patients with hemophilia; NHANES, subjects from the National Health and Nutrition Examination Survey of the general United States population; *b*, regression coefficient for *Z* representing change in log SBP per unit increase of the selected covariate; CI, confidence interval; *r* ^2^, square of the multiple correlation coefficient; BMI, body mass index; eGFR, estimated glomerular filtration rate; HCV, hepatitis C virus.

^*∗*^ *Cage*, *Cage* ^2^, log BMI, eGFR, log TotalCholesterol, diabetes, smoking status, HCV, and race.

**Table 8:** Estimated differences in diastolic BP between PWH and NHANES subjects by race for those models that had a significant interaction *Z∗*race; older subjects (30–79 years) not taking antihypertensive medications.

Outcome	Variables in model	Race	Difference between PWH and NHANES (mmHg)	95% CI
DBP	*Z*, *Cage*, *Cage* ^2^, race, *Z∗*race	White	5.88	3.39, 7.88
		Black	3.04	−2.95, 9.02
		Hispanic	1.76	−2.57, 6.08
		Other	0.80	−4.95, 6.54
DBP	*Z*, *Cage*, *Cage* ^2^, log BMI, eGFR, log TotalCholesterol, smoking status, HCV, race, *Z∗*race	White	4.98	1.13, 8.84
		Black	0.17	−8.55, 8.88
		Hispanic	−0.31	−6.25, 5.63
		Other	0.58	−6.91, 8.07
DBP	*Z*, *Cage*, *Cage* ^2^, log BMI, eGFR, log TotalCholesterol, smoking status, race, *Z∗*race	White	8.43	5.64, 11.22
		Black	3.34	−4.98, 11.66
		Hispanic	1.97	−3.32, 7.26
		Other	3.17	−3.82, 10.17

*Z*  is the binary variable that distinguishes PWH from NHANES (*Z* = 1 for PWH, *Z* = 0 for NHANES); CI indicates confidence interval; *Cage*, centered age; BMI, body mass index; eGFR, estimated glomerular filtration rate; HCV, hepatitis C virus.

**Table 9:** Comparison of the effect of *Z* and HCV on blood pressure models after adjusting for all other covariates.

Outcome	Age-class	Treated?	*P* for *Z*	*P* for HCV
log SBP	18–29	No	<0.001	0.452
log SBP	30–79	No	0.769	<0.001
log SBP	30–79	Yes	0.045	0.668
DBP	18–29	No	<0.001	0.327
DBP	30–79	No	0.905	0.010
DBP	30–79	Yes	0.003	0.466

*Z* is the binary variable that distinguishes PWH from NHANES (*Z* = 1 for PWH, *Z* = 0 for NHANES); HCV, hepatitis C virus.
